# Single-Cell Transcriptome Profiling Reveals the Immune Dysregulation Characteristics of Mice Infected With *Brucella abortus*

**DOI:** 10.1093/infdis/jiaf522

**Published:** 2025-10-11

**Authors:** Guangzhi Zhang, Qingchun Shen, Jianxin Ye, Yu Feng, Pascal Boireau, Xuezheng Fan, Lang Lv, Yan Li, Xiaofeng Xu, Heleer Cha, Chenguang Shen, Yinghui Zhang, Xiaowei Peng, Hui Jiang, Jiabo Ding

**Affiliations:** Animal Biosafety and Public Health Prevention and Control Innovation Team, Institute of Animal Science, Chinese Academy of Agricultural Sciences, Beijing, China; Key Laboratory of Animal Biosafety Risk Prevention and Control (North), Ministry of Agriculture and Rural Affairs (MARA), Institute of Animal Science, Chinese Academy of Agricultural Sciences, Beijing, China; Animal Biosafety and Public Health Prevention and Control Innovation Team, Institute of Animal Science, Chinese Academy of Agricultural Sciences, Beijing, China; Key Laboratory of Animal Biosafety Risk Prevention and Control (North), Ministry of Agriculture and Rural Affairs (MARA), Institute of Animal Science, Chinese Academy of Agricultural Sciences, Beijing, China; Animal Biosafety and Public Health Prevention and Control Innovation Team, Institute of Animal Science, Chinese Academy of Agricultural Sciences, Beijing, China; Key Laboratory of Animal Biosafety Risk Prevention and Control (North), Ministry of Agriculture and Rural Affairs (MARA), Institute of Animal Science, Chinese Academy of Agricultural Sciences, Beijing, China; Jiangsu Key Laboratory of Zoonosis, Yangzhou University, Yangzhou, Jiangsu, China; Animal Biosafety and Public Health Prevention and Control Innovation Team, Institute of Animal Science, Chinese Academy of Agricultural Sciences, Beijing, China; Key Laboratory of Animal Biosafety Risk Prevention and Control (North), Ministry of Agriculture and Rural Affairs (MARA), Institute of Animal Science, Chinese Academy of Agricultural Sciences, Beijing, China; Laboratoire de Santé Animale, Agency for Food, Environmental and Occupational Health & Safety (ANSES), Maisons-Alfort, France; Animal Biosafety and Public Health Prevention and Control Innovation Team, Institute of Animal Science, Chinese Academy of Agricultural Sciences, Beijing, China; Key Laboratory of Animal Biosafety Risk Prevention and Control (North), Ministry of Agriculture and Rural Affairs (MARA), Institute of Animal Science, Chinese Academy of Agricultural Sciences, Beijing, China; Animal Biosafety and Public Health Prevention and Control Innovation Team, Institute of Animal Science, Chinese Academy of Agricultural Sciences, Beijing, China; Key Laboratory of Animal Biosafety Risk Prevention and Control (North), Ministry of Agriculture and Rural Affairs (MARA), Institute of Animal Science, Chinese Academy of Agricultural Sciences, Beijing, China; College of Veterinary Medicine, China Agricultural University, Beijing, China; Animal Biosafety and Public Health Prevention and Control Innovation Team, Institute of Animal Science, Chinese Academy of Agricultural Sciences, Beijing, China; Key Laboratory of Animal Biosafety Risk Prevention and Control (North), Ministry of Agriculture and Rural Affairs (MARA), Institute of Animal Science, Chinese Academy of Agricultural Sciences, Beijing, China; Jiangsu Key Laboratory of Zoonosis, Yangzhou University, Yangzhou, Jiangsu, China; Animal Biosafety and Public Health Prevention and Control Innovation Team, Institute of Animal Science, Chinese Academy of Agricultural Sciences, Beijing, China; Key Laboratory of Animal Biosafety Risk Prevention and Control (North), Ministry of Agriculture and Rural Affairs (MARA), Institute of Animal Science, Chinese Academy of Agricultural Sciences, Beijing, China; School of Public Health, Department of Laboratory Medicine, Southern Medical University, Guangzhou, China; National Reference Laboratory for Animal Brucellosis, China Institute of Veterinary Drug Control, Beijing, China; National Reference Laboratory for Animal Brucellosis, China Institute of Veterinary Drug Control, Beijing, China; Animal Biosafety and Public Health Prevention and Control Innovation Team, Institute of Animal Science, Chinese Academy of Agricultural Sciences, Beijing, China; Key Laboratory of Animal Biosafety Risk Prevention and Control (North), Ministry of Agriculture and Rural Affairs (MARA), Institute of Animal Science, Chinese Academy of Agricultural Sciences, Beijing, China; Animal Biosafety and Public Health Prevention and Control Innovation Team, Institute of Animal Science, Chinese Academy of Agricultural Sciences, Beijing, China; Key Laboratory of Animal Biosafety Risk Prevention and Control (North), Ministry of Agriculture and Rural Affairs (MARA), Institute of Animal Science, Chinese Academy of Agricultural Sciences, Beijing, China

**Keywords:** brucellosis, single-cell RNA sequencing, macrophages, natural killer cells, type I IFN, cell death, NKG2A

## Abstract

**Background:**

Brucellosis poses a significant threat to animal and human health globally. However, how *Brucella* subverts the immune response to establish persistent infections remains unclear.

**Methods:**

We utilized single-cell RNA sequencing (scRNA-seq) to decipher the immune landscape of mice infected with *Brucella abortus*. Flow cytometry, a transgenic cell line and mouse, and antibody blockage were utilized to explore the relevant mechanisms.

**Results:**

*Brucella* infection induced significant changes in the composition and signaling pathways of immune cells, and flow cytometry analysis further confirmed the scRNA-seq data. An in-depth analysis of macrophages, the main target cell for *Brucella*, demonstrated activation of type I interferon (IFN) and type II IFN signaling, tumor necrosis factor production, diverse cell deaths, etc. Specifically, Vir-2308 *Brucella* infection induced IFN-β expression, primarily originating from macrophages. In vitro, a significantly lower level of intracellular *Brucella* survival was observed in *ifnar1*^−/−^ macrophages. In vivo, *ifnar1* genetic deficiency rendered the mice less susceptible to *Brucella* challenge resulting in a lower bacterial load and higher levels of macrophages and neutrophils. Interestingly, *Brucella* infection induced a dramatic reduction of NK cells along with the upregulation of CD94:NKG2A, one typical immune checkpoint module of NK cells. Further blockage of the NKG2A receptor in mice significantly reduced the bacterial load in the tissues, concurrent with a higher ratio of mature dendritic cells and a lower proportion of B cells.

**Conclusions:**

scRNA-seq revealed that *Brucella* infection significantly alters the immune microenvironment in mice, providing insight into a better understanding of brucellosis pathogenesis and the immune evasion strategies of this sophisticated pathogen.

## Teaser

*Brucella* infection profoundly reshapes the immune atlas of mice, providing potential intervention targets against *Brucella* infection.

Brucellosis, a neglected yet widespread zoonotic disease, is caused by facultative intracellular *Brucella* species. Brucellosis can cause abortion, sterility, and other reproductive problems in humans and animals. The disease is characterized in humans by undulant fever, weakness, malaise, and musculoskeletal, cardiac, and neurological complications. In domestic animal, the bacteria induce serious socioeconomic losses worldwide [1–3]. Brucellosis remains endemic in many regions of the world, particularly in developing countries and areas where animal husbandry is rapidly expanding [4–6]. This emphasizes the urgency to reduce this public health burden.

In general, *Brucella abortus* enters the host body predominantly via the mucous membranes of the gastrointestinal tract, respiratory tract, and conjunctiva. At these sites, most bacteria are eliminated through phagocytosis by macrophages (Mϕ) and dendritic cells (DCs); however, some *B. abortus* use diverse immune evasion mechanisms and survive and persistently reside in preferred niches [1, 7]. These mechanisms are not fully elucidated although great efforts have been made [8–11].

Single-cell RNA sequencing (scRNA-seq) has recently been extensively applied to explore host immune responses to certain serious pathogens, including *Mycobacterium tuberculosis*, African swine fever virus, and severe acute respiratory syndrome coronavirus 2 (SARS-CoV-2) [12–16]. The scRNA-seq analysis of immune cells from the infected niche in vivo can aid in revealing the actual immune response pattern during *B. abortus* infection. Understanding the dynamics of immune responses during *Brucella* infection is crucial for improving clinical interventions against brucellosis in humans and animals. Mice serve as a standard model for studying brucellosis in both fundamental research and clinical investigations. However, the immune landscape in murine brucellosis remains poorly characterized. Therefore, we here conducted an scRNA-seq analysis of cells collected from the spleens and lymph nodes of *B. abortus*-infected mice. This is the first study offering a better understanding of the *Brucella*–host interaction using the murine model, especially the immune modulation mechanism exploited by this bacterium. These findings provide valuable insights into *B. abortus* pathogenesis and *Brucella* spp infections, offering reference data for improved brucellosis control in hosts.

## METHODS

### Ethics Statement

The mouse experiment was evaluated and approved by the local ethical committee of China Institute of Veterinary Drug Control (IVDC-2019-056).

### Bacterial Culture and Mice

*B. abortus* strains, vaccine strain A19 (Vac-A19) and virulent strain 2308 (Vir-2308), were cultured and prepared for cell experiments and mouse experiments based on the protocol in the Supplementary Material. *Ifnar1*^−/−^ mice (age, 6–8 weeks) were kindly provided by Professor Wentao Yang from Jilin Agricultural University. All mice were in the C57BL/6 background and were age- and sex-matched for the experiments.

### Experimental Procedure

Mouse infection was performed according to the previous protocol [17]. Detailed information is in the Supplementary Material.

### scRNA Sequencing

Information on scRNA-seq data processing, dimensionality reduction, clustering, cell-type annotation, and differentially expressed genes and functional pathway enrichment is in the Supplementary Material.

### Establishment of *ifnar1*^−/−^ Raw 264.7 Cells Using CRISPR/Cas9

*ifnar1* in murine Mϕ (Raw 264.7 cells) was disrupted using the CRISPR/Cas9-mediated gene editing technique as described elsewhere [18–20], and the detailed protocol is in the Supplementary Material.

### Real-Time PCR Analysis

The mRNA expression levels of genes were determined with SYBR Green-based real-time polymerase chain reaction (PCR) analysis based on the protocol in the Supplementary Material. Table 1 presents the sequence information of primers.

### Histological Analysis

The spleens were collected from the wild-type (WT) mice or *Ifnar1*^−/−^ mice infected with Vir*-*2308 for 2 weeks. They were fixed with 4% paraformaldehyde for 24–48 hours, dehydrated, and paraffin embedded for histological analysis.

### Flow Cytometry Analysis

Tissues of spleens and mesenteric lymph nodes were collected from all mice. The single-cell suspensions of the spleens and lymph nodes were obtained and stained based on the protocol in the Supplementary Material. The detailed information on all the antibodies is summarized in Table 2.

**Table 2.** Antibodies Used for Flow Cytometry Analysis

Antibody Name	Clone No.	Company
Ghost Dye UV450	…	Tonbo
PE-Cyanine7 anti-mouse CD45	30-F11	Tonbo
CD4^+^ PerCP-Cyanine5.5	RM4-5	Tonbo
CD8α APC-Cyanine	53-6.7	Tonbo
F4/80^+^ APC	BM8.1	Tonbo
B220^+^ Alexa Fluor 700 (CD45R)	RA3-6B2	Tonbo
CD3^+^ violetFluor 500	17A2	Tonbo
CD3^+^ Brilliant Ultra Violet 737	17A2	Thermo Fisher Scientific
CD25^+^ violetFluor 450 CD2	PC61.5	Tonbo
CD11B^+^ BV711, Ly-6C FITC anti-mouse	HK1.4	Invitrogen
Brilliant Violet 785 anti-mouse CD11c	N418	Invitrogen
Brilliant Violet 650 anti-mouse I-A/I-E	M5/114.15.2	Biolegend
Brilliant Violet 605 anti-mouse NK-1.1	PK136	Biolegend
PE anti-mouse Foxp3	3G3	Tonbo
PE anti-mouse NKG2AB6	16A11	Biolegend
Anti-mouse NKG2A/C/E Monoclonal Antibody, eBioscience	20D5	Invitrogen

Abbreviations: APC, allophycocyanin; FITC, fluorescein isothiocyanate; PE, phycoerythrin; PerCP, peridinin chlorophyll Protein Complex.

### Blockage of the NKG2A Receptor in Mice by Antibody Administration

The NKG2A receptor in mice was inactivated by antibody administration. A preliminary experiment was first conducted to determine the optimal procedure for NKG2A receptor blockage. Detailed information about the preliminary and formal blockage experiment is in the Supplementary Material. The details of all antibodies are summarized in Table 2.

**Table 1.** Primer Sequences

Primer Name	Primer Sequence, 5′–3′
m GAPDH F	GAGCCAAACGGGTCATCATCT
m GAPDH R	GAGGGGCCATCCACAGTCTT
m IFN-α F	CCTGTGTGATGCAGGAACC
m IFN-α R	TCACCTCCCAGGCACAGA
m IFNb1 F1	AAGAGTTACACTGCCTTTGCCAT
m IFNb1 R1	CACTGTCTGCTGGTGGAGTTCATC

Abbreviations: F, forward; R, reverse; m, mouse.

### Cytokine Determination by ProcartaPlex Multiple Immunoassays

The serum levels of 11 cytokines and chemokines were determined based on the detailed protocol in the Supplementary Material.

### RNA Sequencing

WT Raw 264.7 cells or *ifnar1*^−/−^ Raw 264.7 cells were infected with Vir-2308, and total RNA was extracted for further RNA-seq. The detailed experimental setup is described in the Supplementary Material.

### Statistical Analysis

Graphpad Prism was used for data processing and figure preparation. A Student *t* test was conducted to compare statistical differences between 2 groups. A probability (*p*) value of <0.05 was considered statistically significant.

## RESULTS

### Weights of the Spleen and Lymph Node, and Bacterial Resistance

**Figure 1.**
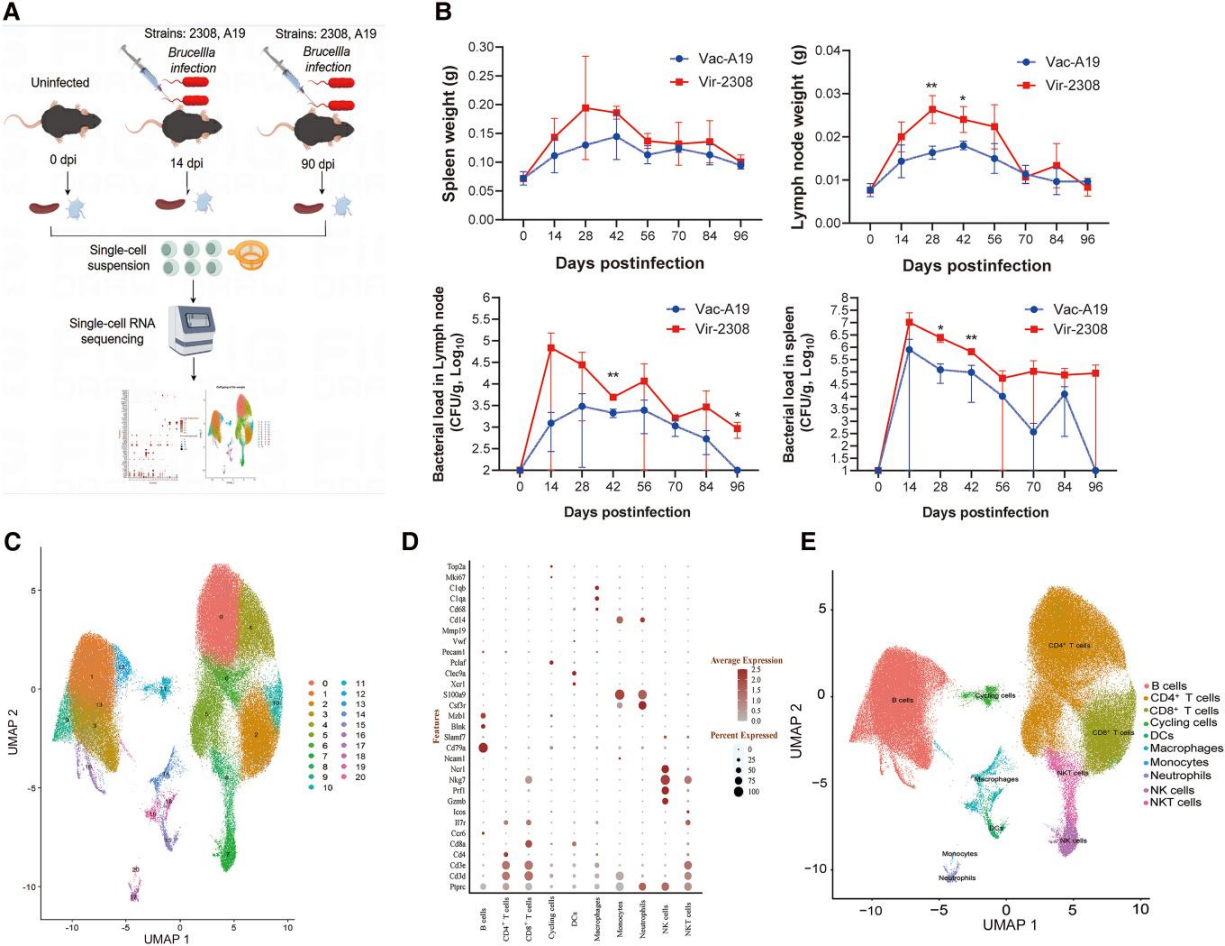
Bacterial load in spleens and inguinal lymph nodes, and organ weight from mice with *Brucella* infection. *A*, The experimental setup for single-cell RNA sequencing. *B*, Weight and bacterial loads of spleen and lymph nodes from mice infected with Vac-A19 or Vir-2308 at 0, 14, 28, 42, 56, 70, 84, and 96 days postinfection. Value represent mean ± SD, *, *p* < 0.05; **, *p* < 0.01. *C*, Totally 21 cell clusters (0-20) were obtained utilizing first-dimension reduction. *D,* Typical marker genes for 10 main cell types. *E*, UMAP was applied to determine the relative proportion of the main cell types. Abbreviations: CFU, colony-forming unit; DC, dendritic cell; dpi, days postinfection; NK, natural killer cell; NKT, natural killer T cell; UMAP, uniform manifold approximation and projection; Vac-A19, *Brucella* vaccine strain A19; Vir-2308, *Brucella* virulent strain 2308 strain. Part of Figure 2*A* was prepared using the https://www.figdraw.com/#/ with some modifications.

Figure 1 *A* presents the workflow experimental setup. Infection with the Vac-A19 or Vir-2308 strain caused splenomegaly and lymphadenectasis in mice from 2 weeks after bacterial inoculation until 84 days postinfection (dpi) (Figure 1*B*). Vir-2308 had a relatively higher effect on the weights of spleens and lymph nodes. The bacterial load of Vir-2308 in both spleens and lymph nodes peaked at 14 dpi and then gradually decreased in both organs. Notably, Vir-2308 persisted in both organs until 96 dpi. By contrast, the bacterial load of Vac-A19 peaked at 14 and 28 dpi in the spleens and lymph nodes, respectively (Figure 1*B*).

### Immune Cell Landscapes in Murine Lymph Nodes

In total, 20 cell clusters were obtained after the first-dimension reduction (Figure 1*C*). Overall, 10 main cell types were annotated using the known typical marker genes, and Figure 1*D* presents cell-specific marker genes for individual cell types. The relative proportion of the 10 major cell types was determined using uniform manifold approximation and projection (UMAP) in the lymph node samples (Figure 1*E*). These cell types chiefly included CD4^+^ T cells, CD8^+^ T cells, B cells, Mϕ, DCs, cycling cells, monocytes, neutrophils (Nϕ), natural killer (NK) cells, and natural killer T (NKT) cells (Figure 1*E*).

**Figure 2.**
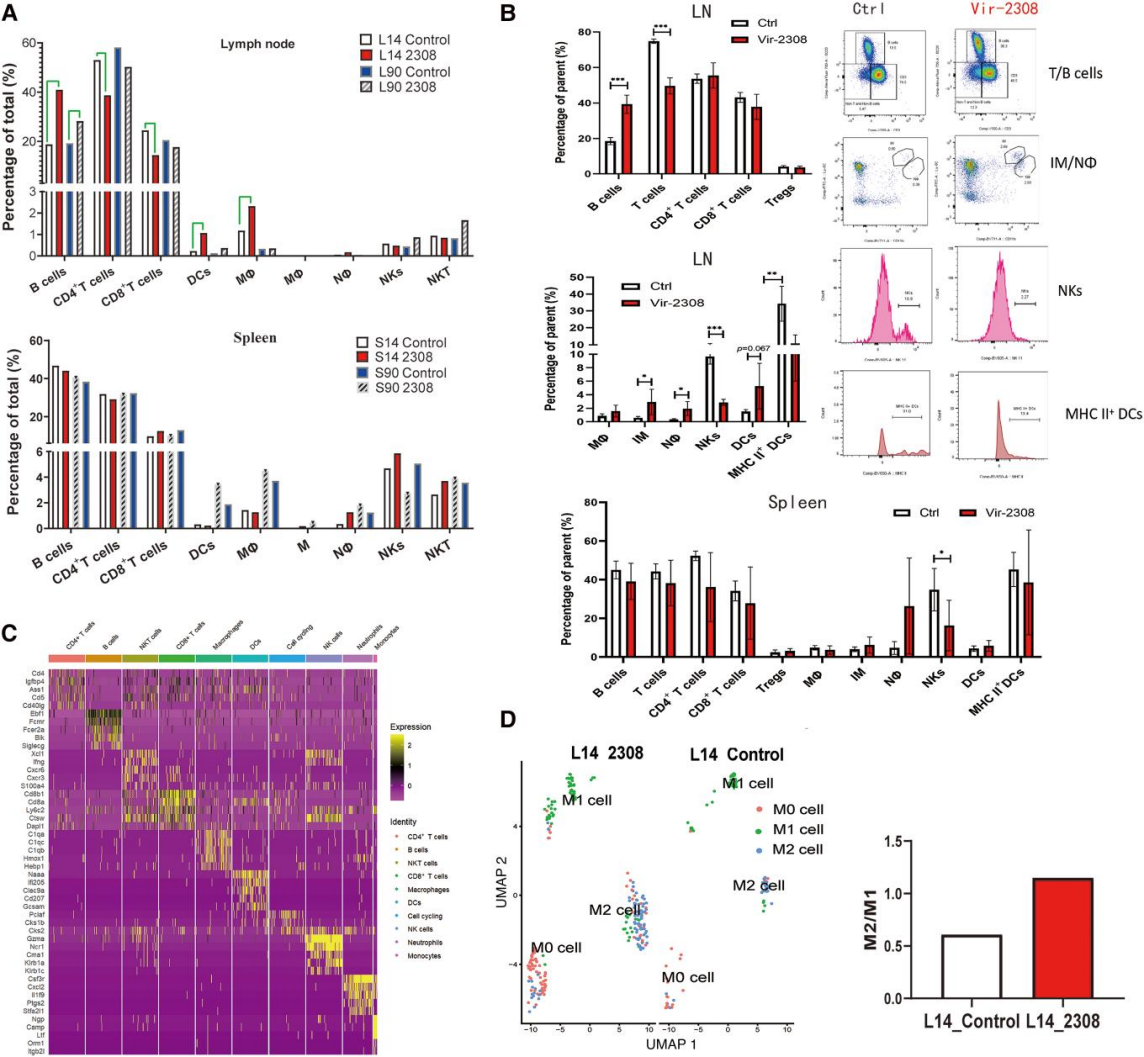
Characterization of immune cell dynamics in response to *Brucella* infection. *A*, The immune cell proportion in the LN and spleens from all experiment groups analyzed by scRNA-seq. *B*, Verification of immune cell (B cells, T cells, CD4^+^ T cells, CD8^+^ T cells, Tregs, Mϕ, IM, Nϕ, DCs, and NKs) ratios in the LN and spleens by flow cytometry analysis. *C*, Heatmap showing the top 5 differentially expressed genes in the 10 main immune cell types examined in the lymph nodes of the Vir-2308 group at 14 days postinfection. *D*, UMAP plots of the 3 cell subtypes (M0, M1, M2) of Mϕ, and histograms of the relative ratio of individual clusters of Mϕ. Value represent mean ± SD. *, *p* < 0.05; **, *p* < 0.01; ***, *p* < 0.001.Abbreviations: L14, lymph nodes from control group or infected group at 14 days post infection; L90, lymph nodes from control group or infected group at 90 days post infection; 2308, *Brucella* 2308 strain; Ctrl, control group; DC, dendritic cells; IM, inflammatory monocytes; LN, lymph nodes; Mϕ, macrophages; NKs, natural killer cells; Nϕ, neutrophils; scRNA, single-cell RNA sequencing; Tregs, regulatory T cells; UMAP, uniform manifold approximation and projection.

Approximately 1.48-fold increased Mϕ was observed in the Vir-2308 group (2.32%) compared with the control group (1.56%) at 14 dpi. A similar but mild trend was observed at 90 dpi. The relative ratio of NK cells decreased in the Vir-2308 group (0.48%) compared with the control group (0.58%). The UMAP plots revealed the Mϕ cell subtypes in the control group and Vir-2308 group (Figure 2*D*). An increased M2:M1 ratio in the lymph nodes was observed in the Vir-2308 group (1.15) at 14 dpi compared with the control group (0.61). Interestingly, compared with the relative ratio of total B cells in the control group (18.80%), the relative ratio of total B cells dramatically increased in the lymph nodes of the Vir-2308 group (40.96%) at 14 dpi, and the same trend but to a less extent was observed at 90 dpi (Figure 2*A*). The changes in DC, Nϕ, and T cells are shown in the Supplementary Material. Flow cytometry data obtained at 14 dpi validated the majority of the scRNA-seq results (Figure 2*B*); the results are included in the Supplementary Material. The gating strategy for flow cytometry analysis is presented in Supplementary File 1.

### Immune Responses and Inflammatory Responses Are Activated in Certain Innate Immune Cells in Response to *Brucella* Infection

**Figure 3.**
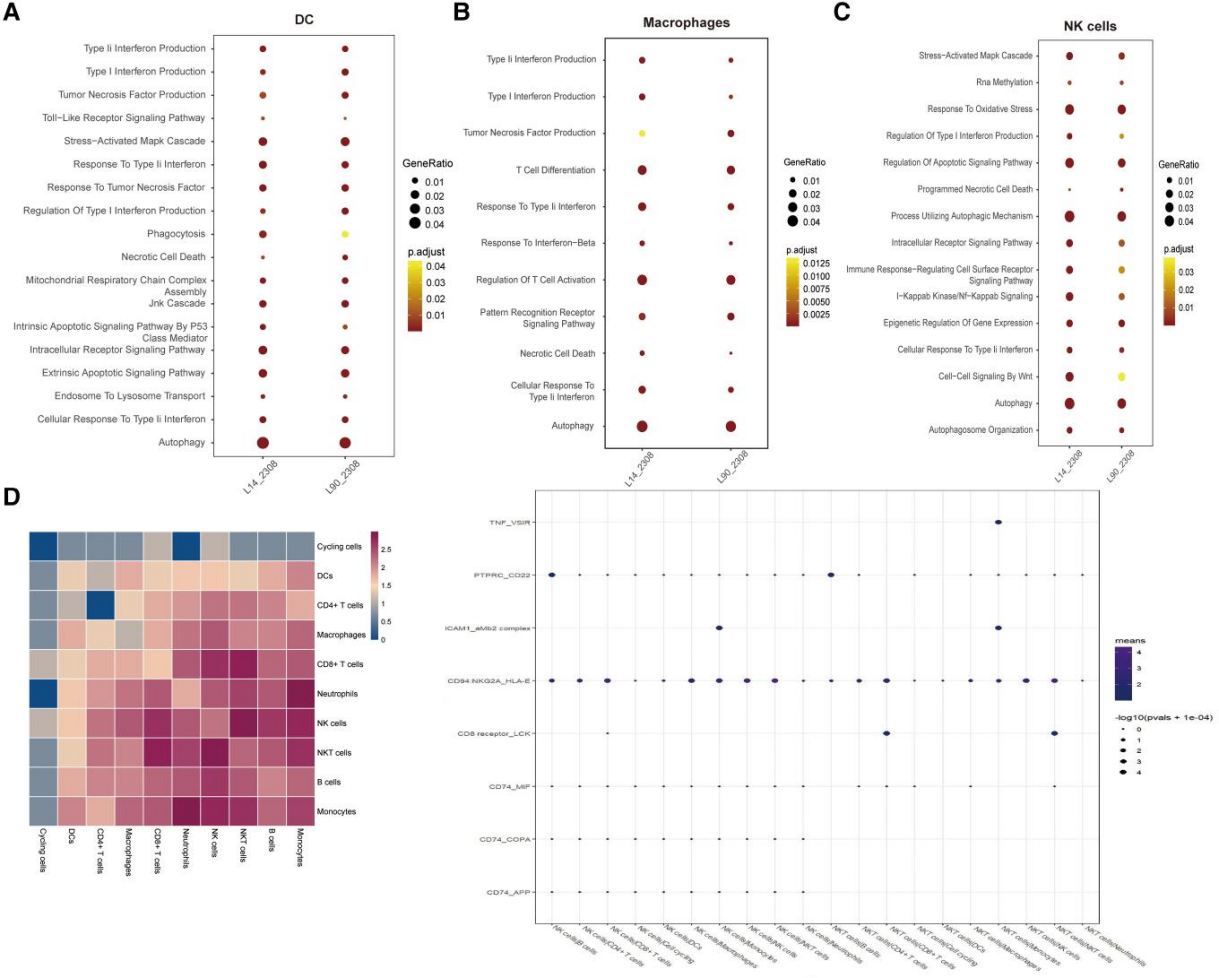
Characterization of host signaling pathways and host genetic changes in response to *Brucella* infection. *A*, Shared hallmark signaling pathways in DCs in the lymph nodes from mice infected with *Brucella* Vir-2308 at 14 and 90 dpi (termed L14_2308 and L90_2308, respectively) are shown as bubble diagrams. *B*, Common hallmark signaling pathways in Mϕ in the lymph nodes infected with Vir-2308 at 14 and 90 dpi. *C*, Common hallmark signaling pathways in NKs in the lymph nodes from mice infected with Vir-2308 at 14 and 90 dpi. In the bubble histograms, dot size indicates the gene expression ratio, and dot color indicates adjusted *P* values. *D*, Left, Cell communication analyzed using CellphoneDB revealed the cell–cell interaction profile between 10 main types of immune cells in lymph nodes (B cells, CD4^+^ T cells, CD8^+^ T cells, Mϕ, IM, Nϕ, DCs, NKs, NKT). Right, selected ligand–receptor interactions in 10 types of immune cells in lymph nodes based on CellphoneDB. Dot size indicates *P* values, dot color indicates the means of the receptor–ligand pairs between 2 cell clusters, and color scale represents the interaction strength between cells. Abbreviations: DC, dendritic cells; dpi, days postinfection; IM, inflammatory monocytes; Mϕ, macrophages; NKs, natural killer cells; NKT, natural killer T cells; Nϕ, neutrophils.

In brief, we present a heatmap depicting the top 5 differentially expressed genes (DEGs) and enriched Kyoto Encyclopedia of Genes and Genomes (KEGG) pathways of several cell clusters; the heatmap shows the top 5 DEGs in the lymph nodes of the Vir-2308 group at 14 dpi (Figure 2*C*). Moreover, type I and type II interferon (IFN) production, tumor necrosis factor (TNF) production, T-cell differentiation, responses to IFN, regulation of T-cell activation, pattern recognition receptor signaling pathway, necrotic cell death, and autophagy were activated in Mϕ in the lymph nodes of Vir-2308 group at both time points (Figure 3*B*). The shared signaling pathways (eg, MAPK cascade, RNA methylation, responses to oxidative response, type II IFN production, cell apoptosis, epigenetic regulation of gene expression, and Wnt signaling) were enriched in the NK cells in the Vir-2308 groups at 14 and 90 dpi (Figure 3*C*). A series of inflammatory pathways and immune signaling pathways were elicited in Mϕ. Moreover, we discovered that *Brucella* infection caused a sharp decline in NK cells, and diverse cell death pathways were also induced in NK cells. The enriched pathways in the Mϕ, Nϕ, DCs, and NK cells are summarized in Supplementary Files 2–6. Vir-2308 infection elicited strong immune responses in mice compared with Vac-A19 infection (Supplementary File 7).

### IFN Signaling Pathways, Cell Death Pathways, and Certain Immune Signaling Pathways Were Activated in Mice in Response to *Brucella* Infection

The scRNA-seq data revealed that *Brucella* infection, especially that caused by Vir-2308, activated type I and type II IFN signaling and cell death pathways, compared with the control group (Supplementary File 8*A*–*C*). Overall, type II IFN (IFN-γ) was induced at a relatively high level during *Brucella* infection, especially in NK cells and NKT cells (Supplementary File 8*A*). Higher expression levels of IFN-γ were seen in NK cells from both spleens and lymph nodes from the Vir-2308 group and the Vac-A19 group at 14 dpi, especially in the Vac-A19 group (Supplementary File 8*H*). Moreover, elevated expression levels of IFN-γ were seen in NKT cells in spleens and lymph nodes at 90 dpi, with the highest level in spleens from the Vir-2308 group at 90 dpi (Supplementary File 8*I*). Also, higher expression levels of IFN-γ were observed in CD8^+^ T cells in the spleens from the Vir-2308 group at 90 dpi (Supplementary File 8*J*). There were no obvious changes in IFN-γ expression in CD4^+^ T cells from spleens and lymph nodes (Supplementary File 8*K*). The expression profile of the IFN-γ receptor, *Ifngr1*, exhibited a similar trend. Mϕ was the main source of type I IFN (IFN-β) production in the *Brucella*-infected mice (Supplementary File 8*D*). Moreover, IFN-β was the main type I IFN produced in mice in response to virulent *Brucella* infection, and *IFNb1* expression was mainly observed in the lymph nodes of the Vir-2308 group at 14 dpi (Supplementary File 8*E*), and lymph nodes in the Vir-2308 group exhibited a relatively higher level of type I IFN-related regulatory genes, such as *Irf3*, *Irf7*, and *IrfF9*, compared with that in the control group (Supplementary File 8*B*). Changes in cell death signaling pathway-related genes are presented in the Supplementary Material.

Our data showed that CD94:NKG2A_HLA-E is induced in NK and NKT cells during *Brucella* infection (Figure 3*D*). CD94:NKG2A, representing one typical immune checkpoint module, limits the antitumor or antiviral activities of NK cells [21, 22]. More detailed strong ligand–receptor interactions are listed in Supplementary File 9.

### Type I IFN Signaling Contributes to *Brucella* Infection Pathogenesis

**Figure 4.**
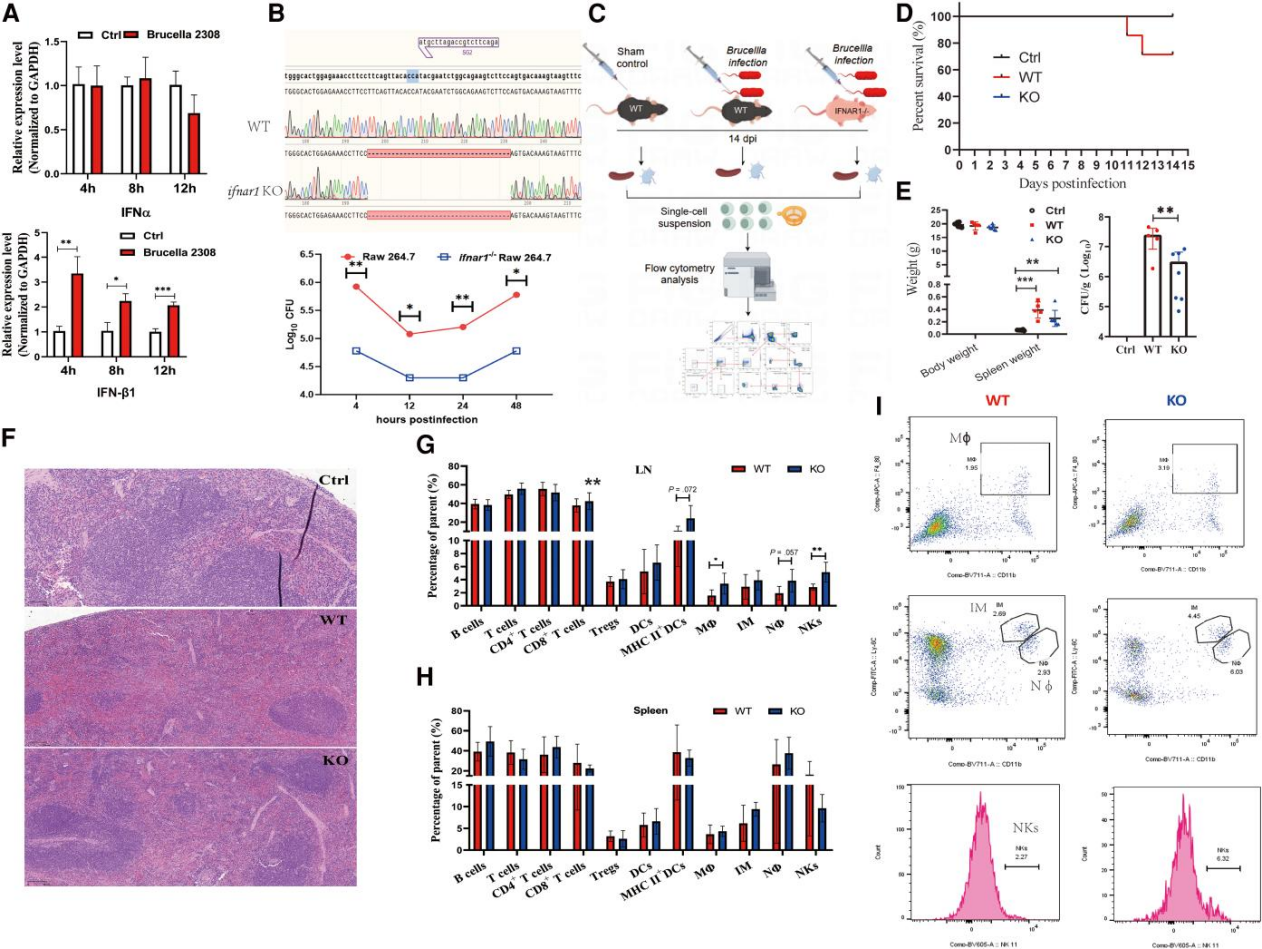
Type I IFN signaling contributes to the pathogenesis of *Brucella* infection in murine macrophages (Raw 264.7 cells) and mice. WT mice and *Ifnar1*^−/−^ mice were infected with *Brucella* 2308 for 2 weeks, spleens and lymph nodes were collected, and a series of parameters were determined. *A*, Real-time PCR showed that *Brucella* 2308 infection induced elevated expression of IFN-β in Raw 264.7 cells. *B*, *Ifnar1*^−/−^ Raw 264.7 cells were established using the CRISPR/Cas9 technique, and the gene editing efficacy was verified by PCR and sequencing. The intracellular survival of *Brucella* 2308 was determined in WT Raw 264.7 cells and *Ifnar1*^−/−^ Raw 264.7 cells at 4, 12, 24, 48 hours postinfection. *C,* Schematic illustration of the experimental workflow for exploring the role of *ifnar1* during *Brucella* infection in mice. *D*, Survival curve of the control mice, and *Brucella*-infected WT mice or *ifnar1*^−/−^ mice. *E*, Weight of the body and spleens collected from mice infected with *Brucella* (left). The bacterial load of *Brucella* in the spleens from control mice, *Brucella*-infected WT mice, and *ifnar1*^−/−^ mice are presented (right). *F*, Hematoxylin and eosin staining of spleen samples from the control, infected WT mice, and infected *Ifnar1*^−/−^ mice group (KO). *G* and *H*, The relative abundance of 11 cell types (B cells, T cells, CD4^+^ T cells, CD8^+^ T cells, Tregs, Mϕ, IM, Nϕ, DCs, MHC II^+^ DCs, NKs) in the lymph nodes (*G*) and spleens (*H*) was determined by flow cytometry. *I*, Flow cytometry dot plot and histograms of representative images of Mϕ, IM, Nϕ, and NKs in *Brucella*-infected WT mice or *ifnar1*^−/−^ mice. Value represent mean ± SD. *, *p* < 0.05; **, *p* < 0.01; ***, *p* < 0.001. Abbreviations: CFU, colony-forming unit; Ctrl, control; DCs, dendritic cells; dpi, days postinfection; GAPDH, glyceraldehyde-3-phosphate dehydrogenase; IFN, interferon; IM, inflammatory monocytes; LN, lymph nodes; Mϕ, macrophages; NKs, natural killer cells; Nϕ, neutrophils; PCR, polymerase chain reaction; Tregs, T regulatory cells; Ctrl, control group without *Brucella* infection; WT, wild type; KO, Knockout. Part of Figure 4*C* was prepared using the https://www.figdraw.com/#/ with some modifications.

The IFN-β transcriptional expression level significantly increased in the Vir-2308-infected Raw 264.7 cells at 4, 8, and 12 hours postinfection (hpi) (Figure 4*A*). Genetic disruption of *ifnar1* in Raw 264.7 cells was completed and confirmed through PCR and gene sequencing (Figure 4*B*). Intracellular survival of *Brucella* was lower in *ifnar1*^−/−^ Raw 264.7 cells at 4, 12, 24, and 48 hpi than in WT Raw 264.7 cells (Figure 4*B*). RNA-seq analysis further showed that, compared to Vir-2308–infected WT Raw 264.7 cells, 547 upregulated genes and 1435 downregulated genes were observed in *ifnar1*^−/−^ Raw 264.7 cells infected with Vir-2308 (Supplementary File 10). Go analysis demonstrated that significantly enriched pathways mainly included immune response, response to type I and type II IFN, protein binding, phagocytosis, and other immune or inflammation relevant pathways. Notably, a series of phagocytosis-related genes (eg, *CD36*, *ITGB3*, *TYRO3*, *CCR2*, *TREX1*, *UNC13D*) were significantly downregulated upon *Ifnar1* inactivation, suggesting impaired phagocytic function in Raw 264.7 cells in the current setting of *Brucella* infection (Supplementary File 11).

*Ifnar1^−^* ^/*−*^ mice and littermate control mice were subjected to an inguinal subcutaneous challenge with 1 × 10^5^ colony-forming units Vir-2308 (Figure 4*C*, 4*D*). Notably, a significantly lower bacterial load (*P* < .01) was observed in the spleen from the *Brucella*-infected *Ifnar1*^−/−^ mice compared with the *Brucella*-infected WT mice (Figure 4*E*). Histopathological changes (Figure 4*F*) and immune cell alterations are shown (Figure 4*G* –4*I*) in the Supplementary Material.

### Blockage of the NKG2A Receptor Facilitates the Clearance of *Brucella* in Mice

**Figure 5.**
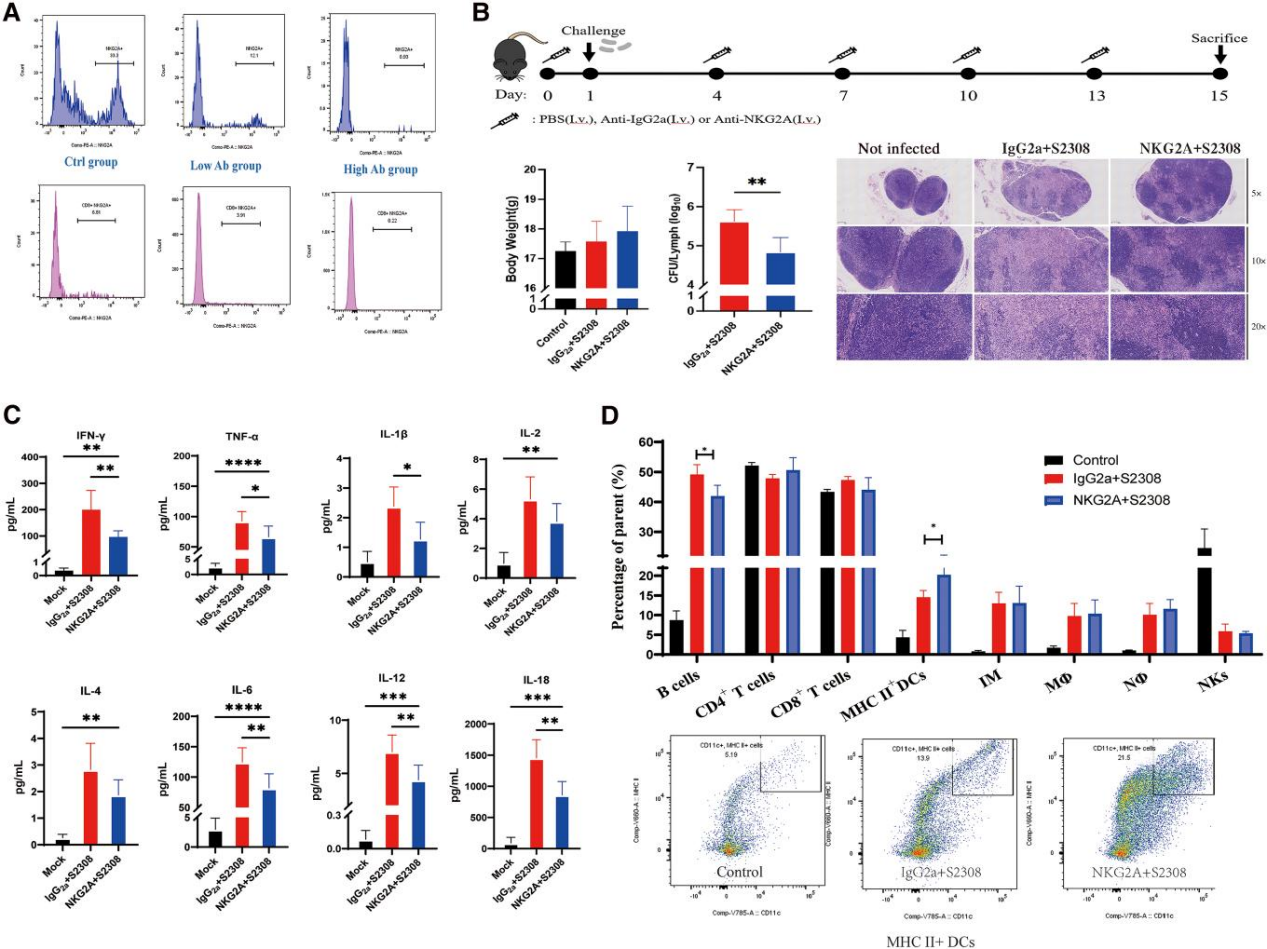
NKG2A blockage contributes to the clearance of *Brucella* in mice. *A*, Administration of 100 µg anti-mouse NKG2A/C/E antibody through tail veil injection was given at 3-day intervals (high Ab group) or 5-day intervals (low Ab group). The 3-day intervals exhibited good blockage efficacy in NK cells and CD8^+^ T cells from mice. *B*, Schematic showing the experimental setup for evaluating the effects of NKG2A blockage on *Brucella* infection in 3 groups of mice: control group (noninfection group), IgG2a + S2308 group (antibody isotype control-treated mice challenged with *Brucella* 2308), NKG2A + S2308 group (anti-mouse NKG2A/C/E antibody-treated mice challenged with *Brucella* 2308). The body weight of the mice, and the bacterial load in the lymph nodes from isotype control group and NKG2A blockage group are shown. Hematoxylin and eosin staining of lymph node samples from the control group, IgG2a + S2308 group, and NKG2A + S2308 group. *C*, Serum was collected from mice inoculated with the isotype control antibody and anti-mouse NKG2A/C/E antibody, and cytokines levels (IFN-γ, IL-1β, IL-2, IL-4, IL-6, IL-12, IL-18, TNF-α) were assessed by ProcartaPlex multiple immunoassays. *D*, Flow cytometry analysis revealed the effects of NKG2A blockage on the key immune cell populations (B cells, CD4^+^ T cells, CD8^+^ T cells, MHC II^+^ DCs, inflammatory monocytes, Mϕ, Nϕ, and NK cells) in the lymph nodes of mice. The representative flow cytometry images gating the MHC II^+^ DCs in the lymph nodes from 3 groups are presented. Value represent mean ± SD. *, *p* < 0.05; **, *p* < 0.01; *, *p* < 0.001.Abbreviations: S2308, *Brucella* virulent strain 2308 strain; CFU, colony-forming unit; Ab, antibody; DCs, dendritic cells; IFN-γ, interferon-γ; IL, interleukin; IV, intravenous; Mϕ, macrophages; NK, natural killer cells; Nϕ, neutrophils; PBS, phosphate-buffered saline; TNF-α, tumor necrosis factor-α.

Flow cytometry data showed that administration of 100 µg anti-mouse NKG2A/C/E antibody through tail veil injection at 3-day intervals exhibited satisfactory NKG2A blockage in both NK cells and CD8^+^ T cells (Figure 5*A*). Compared with the isotype control treatment group, NKG2A blockage significantly decreased the survival of *Brucella* in the lymph nodes of mice (Figure 5*B*). Histopathological analysis showed that *Brucella* infection resulted in fewer number of lymphonoduli through their destruction, and NKG2A blockage reversed this phenotype, resulting in a normal number and structure of lymph nodes(Figure 5*B*). Compared with the isotype control group, significantly lower levels of several proinflammatory cytokines, including IFN-γ, IL-1β, IL-6, IL-12 p70, IL-18, and TNF-α, were observed in the NKG2A blockage group in response to *Brucella* infection (Figure 5*C*). Only a significantly higher level of MHC II^+^ DCs and a lower level of B cells were observed in the NKG2A blockage group compared with the isotype control group (Figure 5*D*).

## DISCUSSION

Although extensive efforts have been made, the exact mechanism through which *Brucella* manipulates host immune responses remains largely unclear [23–25]. Our scRNA-seq data demonstrated that *Brucella* infection reorganizes the immune microenvironment of the lymph node, characterized by dramatic loss of NK cells and T cells, and elevated levels of IM, Nϕ, and B cells. Recently, a group from China utilized scRNA-seq to analyze the immune landscape of peripheral blood mononuclear cells (PBMCs) from patients with brucellosis; they discovered that cytokine storm is the typical characteristic of acute *Brucella* infection, and T cell and NK cell exhaustion [26].

The distinct roles of type I IFN in different bacterial infections have been observed [27–29]. A few studies have reported the regulatory role of type I IFN in response to *Brucella* infection. Alonso Paiva et al demonstrated that respiratory *B. abortus* infection in mice induced higher IFN-β mRNA expression levels in the lung and spleen in a stimulator of interferon genes (STING)-dependent manner, and a lower bacterial load was observed in the STING knockout mice [30]. In another study, *Brucella* Omp25 inhibited IFN-β production induced by various DNA viruses or IFN-stimulatory DNA in monocytes/Mϕ [31]. Our *in vitro* experiment revealed that the intracellular survival ability of *Brucella* was significantly compromised in *Ifnar1*^−/−^ murine Mϕ compared with WT Mϕ. Furthermore, RNA-seq analysis demonstrated that a series of signaling pathways (eg, phagocytosis, regulation of phagocytosis, response to interferon-beta, protein biding, etc.) were enriched in *Ifnar1^−/−^* Raw 264.7 Mϕ infected with *Brucella*. Notably, the phagocytosis-associated signaling pathway was downregulated in *Brucella*-infected *Ifnar1^−/−^* Raw 264.7 Mϕ compared with the WT group, which may partially account for the decrease of intracellular bacteria. The transgenic mouse model demonstrated that *Ifnar1* deficiency in mice promoted *Brucella* elimination, possibly by recruiting several key innate immune cells. Because IFN-γ is a key cytokine against intracellular bacterial infection, some pathogens develop diverse strategies to attenuate IFN-γ signaling and promote host susceptibility, for example, by inhibiting the IFN-γ receptor [29]. Here, we did not explore the relationship between type I IFN and type II IFN during *Brucella* infection in hosts. The exact mechanisms through which type I IFN promotes host susceptibility to *Brucella* infection await further investigation. Our data also demonstrated that *Brucella* infection could induce high expression levels of *ifng* in the spleen compared to the control group, a phenomenon we also consistently observed in Mϕ. Regarding the elevated basal expression of *ifng* in the control spleen group, we speculate that certain unknown factors may lead to the increased basal expression of *Ifng*.

Studies of *Brucella* and NK cell interaction are limited. Transcriptomic analysis with PBMCs infected with *B. abortus* RB51 revealed several downregulated genes, including those related to NK cell-mediated immune responses [32]. Moley et al revealed that NK cell-deficient mice developed more severe arthritis, characterized by lower IFN-γ levels in their joints, after footpad infection with *Brucella melitensis* 16M [33]. This indicated that NK cells contribute significantly to host defense against *Brucella* infection. Our study showed a clear reduction in NK cells in the lymph nodes of the Vir-2308–infected WT mice. *Brucella* infection may cause massive death of NK cells, because enriched cell death signaling pathways were observed in these mice (eg, programmed necrosis, autophagy). Interestingly, *ifnar1* deficiency in the mice reversed the decrease in the amount of NK cells in the *Brucella*-infected WT mice, suggesting the potential regulatory role of type I IFN in NK cell recruitment and functions during this process.

The contribution of B cells during *Brucella* infection remains undefined. Notably, our scRNA-seq and flow cytometry analyses demonstrated that the B-cell proportion strikingly increased in the lymph nodes of infected mice. Some reports have shown that B cells compromise the host defense against *Brucella* infection in mice. Goenka et al proved that B-cell deficiency conferred increased resistance in mice against *Brucella* infection; this phenotype coincided with elevated levels of IFN-γ^+^ CD4^+^ T cells and CD8^+^ T cells, indicating that B cells potentially interacted with T cells [34]. Indeed, Dadelahi et al found that B cells restrained the CD4^+^ T-cell–mediated protective immune response against *Brucella* [35], which indicates an interaction between B cells and CD4^+^ T cells during murine brucellosis. Similarly, our cell–cell communication analysis revealed that interactions between B cells and CD4^+^ T cells were strong during *Brucella* infection. However, some groups have demonstrated that B cells do not play a significant role in *Brucella*-infected mice. According to Lacey et al, adaptive immune cells, T cells, and B cells are dispensable for the induction of focal inflammation in *Brucella*-infected mice, whereas CXCR2-mediated Nϕ recruitment is indispensable during this process [36]. The reasons for the diverse contributions of B cells during *Brucella* infection are multifaceted, including the host, infection dose, infection period, and infection route. Demars et al demonstrated that different infection routes significantly affect the host–pathogen interaction; according to these authors, the CD4^+^ T-cell–mediated Th1 immune response plays a pivotal role in controlling *Brucella* infection in mice via the intraperitoneal route, while it is dispensable in the intranasal model [37]. Collectively, the modulatory role and functions of B cells in *Brucella* infection need to be further investigated.

Importantly, the functions of NK cells are determined on the basis of the integrated activities of both activating (such as NKG2C and NKG2E) and inhibitory receptors (NKG2A), which are recognized by ligands induced by stress and pathogenic infections [38]. In response to *Brucella* infection, the expression level of CD94-NKG2A, a typical immune checkpoint inhibitor, was elevated in the NK cells. In some cases, inhibitory receptors like NKG2A restrain the activities of antimicrobial responses, and CD94-NKG2A heterodimers inhibit the CD8^+^ T-cell cytotoxic response to polyoma virus [39]. By contrast, mice with NKG2A genetic ablation succumbed to the lethal ectromelia virus, because of the preservation of virus-specific CD8^+^ T cells by NKG2A [40]. Mice with the NKG2C or NKG2E deficiency were resistant to lethal poxvirus infection [41]. The interaction of CD94-NKG2A and ligands (HLA-E in humans, and Qa-1^b^ in mice) may mediate the immune signal of NKs during *Brucella* infection. Indeed, our data demonstrated that a significantly lower level of survival of *Brucella* was observed in the mice when the NKG2A receptor was blocked by antibody administration before the bacterial challenge. The blockage of NAG2A may synergize with the current therapy regimen comprising ceftriaxone, doxycycline, and rifampicin, contributing to an improved efficacy during human brucellosis and animal brucellosis, which needs to be explored in the future. Apparently, the NKG2A blockage in mice does not affect the composition of key immune cell types, except that a higher level of mature DCs and a lower level of B cells were observed. The higher level of mature DCs may mediate a stronger antigen-processing and presentation ability *in vivo*. Whether NKG2A blockage regulates the functions of other cell types (eg, NKs, Mϕ, and CD8^+^ T cells) remains to be further explored. Cell–cell communication analysis demonstrated several strong interactions between NKs and Mϕ or CD8^+^ T cells, indicating the potential effects of NKs on these cell types under the current experimental conditions. Moreover, further in-depth investigations need to be conducted to clarify the role of NK cells in response to *Brucella* infection and the underlying mechanisms, for example, the mechanism of NK exhaustion regulated by *Brucella*.

In summary, our study systematically explored the immune landscape of mice infected with the *Brucella* virulent strain or vaccine strain, in particular revealing the characteristics of immune regulation and immune evasion in brucellosis. *Brucella* may evade the immune system and establish chronic infection in hosts by manipulating host factors, IFN-β and NKG2A. This finding deepens the understanding of brucellosis pathogenesis and providing insights into the treatment of this reemerging worldwide bacterial zoonosis.

## Supplementary Material

jiaf522_Supplementary_Data

## Supplementary Data

Supplementary materials are available at *The Journal of Infectious Diseases* online (http://jid.oxfordjournals.org/). Supplementary materials consist of data provided by the author that are published to benefit the reader. The posted materials are not copyedited. The contents of all supplementary data are the sole responsibility of the authors. Questions or messages regarding errors should be addressed to the author.
