# Supplementary material for: Single-Cell Transcriptome Profiling Reveals the Immune Dysregulation Characteristics of Mice Infected With Brucella abortus
Source: J Infect Dis. 2025 Oct 11;233(1):e55–66. doi: 10.1093/infdis/jiaf522 (PMC12811882; doi:10.1093/infdis/jiaf522)
Supplement: jiaf522_Supplementary_Data [file jiaf522_supplementary_data.zip › Supplementary Materials (Revised)_09262025.docx]

**Methods**

**Bacterial culture and mice**

*B. abortus* strains, vaccine strain A19 (Vac-A19) and virulent strain 2308 (Vir-2308), were obtained from the Chinese Veterinary Culture Collection Center (Beijing, China). The strains were grown on tryptic soy broth (TSB, BD company) at 37°C or on tryptic soy agar at 37°C under 5% CO_2_. The bacterial concentration was determined through plate counting. Specific-pathogen-free C57BL/6 mice (n = 63, gender: female, age: 6–8 weeks) were purchased from Beijing Vital River Laboratory Animal Technology Co., Ltd and randomized into three groups. All mice were allowed to acclimatize at biosafety level III facilities of the China Institute of Veterinary Drug Control for 1 week before they were subjected to any further challenge.

*Ifnar1^−^*^/^*^−^* mice (age: 6–8 weeks) were kindly provided by Prof. Wentao Yang from Jilin Agricultural University. All mice on the C57BL/6 background and age- and gender-matched were selected for the experiment.

**Experimental procedure**

The mouse infection was performed according to the previous protocol [16]. In brief, the mice from the first group received an inguinal subcutaneous inoculation of the medium and served as the control group (Ctrl). The mice from second group (Vac-A19) and third group (Vir-2308) received an inguinal subcutaneous inoculation of with 1 × 10^5^ CFU of Vac-A19 or Vir-2308 with a volume of 100 μL, respectively. Three mice from each group were anesthetized with CO_2_, followed by cervical dislocation at 14, 28, 42, 56, 70, 84, and 96 days post-infection (dpi). The spleen and inguinal lymph nodes from both sides of the mice were collected, weighed, and smashed to produce a single-cell suspension with peptone saline. Then, 100 μL aliquot from the 10-fold serially diluted cell suspension from each mouse was plated on the TSB plate in duplicate. The plates were incubated at 37°C for up to 3 days. The bacterial load in the spleen and lymph node was calculated as CFU/g.

Moreover, at 14 and 96 dpi after the challenge, single-cell suspensions from the spleen or inguinal lymph nodes from each group were pooled together, respectively. The cell clusters or aggregates in the cell suspension were removed using 40-μm cell strainers, and red blood cells were eliminated using 1× Red Blood Cell Lysis buffer (Tonbo Biosciences, San Diego, CA, USA), followed by scRNA-seq.

**scRNA-seq**

Approximately 10,000 cells of the prepared single-cell suspension were barcoded and processed with the 10× Genomics Chromium v2.0 platform using default parameters. cDNA was amplified, followed by library construction. The samples are sequenced on an Illumina NovaSeq 6000 sequencing system (paired-end multiplexing run, 150 bp) at LC-Bio Technology Co. Ltd.

**scRNA-seq data processing**

Original FASTQ files generated from the spleen or lymph node samples were processed using the Cell Ranger (v.6.1.1) count pipeline coupled with the mouse reference version GRCm38 to generate feature-barcode matrices. The Seurat object list was obtained using the Seurat package (S4) with R software (v.4.3.2) and the following criteria: (1) min.cells = 300; (2) 100 < nFeature_RNA < 6000; (3) percent.mt < 10; and (4) percent_hb < 1.5. Additionally, DoubletFinder was applied to eliminate potential doublets in all cells.

**Dimensionality reduction, clustering, and cell-type annotation**

The canonical correlation analysis method was used to decrease batch effects across the samples [17]. In brief, NormalizeData was employed to normalize the filtered gene expression data by using a formula: Gene expression level = log (1 + (UMIA/UMI Total) X10000). Then, the ‘vst’ method with the FindVariableFeatures function was used to identify the top 2,000 variable genes. At last, FindIntegrationAnchors and IntegrateData were employed with the top 20 dimensions within 12 samples. The RunPCA function was applied on linear-transformation scaled data with 2,000 variable features, and tSNE was performed with the top 20 dimensions. The cells were finally clustered using FindNeighbors and FindClusters (resolution = 0.8) functions.

The Seurat FindAllMarkers function was employed to identify cluster markers by using default settings. Typical cell markers for individual cell type were then applied for cluster annotation. The cluster annotation was then confirmed using the R package SingleR with mouse BlueprintEncodeData dataset. In brief, the FindClusters function was used with the resolution setting as 0.1 for Mϕ, DCs, B cells, T cells, and NK cells.

**Differentially expressed genes and functional pathway enrichment**

Differentially expressed genes (DEGs) in all samples were identified using the FindMarkers function in Seurat between different groups with default parameters (logfc.threshold = XXX, test.use = ‘‘wilcox,’’ min.pct = XX). Using the gene ontology (GO) database (http://geneontology.org/) or Reactome pathway database (<https://reactome.org/>), functional analysis was performed with the enrichPathway function in the clusterProfiler package (v.3.15.2).

**Establishment of** ***ifnar1*^-/-^ Raw 264.7 cells using CRISPR/Cas9**

*ifnar1* in murine macrophages (Raw 264.7 cells) was disrupted using the CRISPR/Cas9-mediated gene editing technique as described elsewhere [18, 19]. In brief, two sgRNAs were designed using the UC Santa Cruz optimized CRISPR design tool (https://crispor.gi.ucsc.edu/crispor.py). The sgRNA-targeting murine *ifnar1* was designed based on the report of Blomberg *et al*. [20], and the sgRNA sequences were 5′-ATGTTCCCGTCTTGTCCGGG-3′ and 5′-AGACTTCTGCCAGATTCGTA-3′. sgRNA was synthesized and cloned into the pGL3-U6-sgRNA-GFP plasmid (A kind gift from Huang Xingxu’s lab, Shanghai Tech University, China). The transfection agent, JetPrime (Polyplus, France), was used to introduce all components into HEK293T and assemble the lentivirus. The lentivirus was used to transfect Cas9^+^ Raw 264.7 cells engineered by our lab. The single colony of *ifnar1*^-/-^ Raw 264.7 cells was obtained through flow cytometry sorting and further cultured in a 96-well plate for expansion. *ifnar1^-/-^* disruption in Raw 264.7 cells was confirmed through genomic sequencing.

To study the role of *ifnar1* in *Brucella*-infected Raw 264.7 cells, the intracellular survival of *Brucella* Vir-2308 in WT Raw 264.7 cells and *ifnar1^-/-^* Raw 264.7 cells was determined. In brief, the cells were seeded in 24-well plates (2.5 × 10⁵ cells/mL/well) with 3 replicates in each group and cultured at 37°C under 5% CO_2_ prior to infection. The Raw 264.7 cells were infected by *Brucella* Vir-2308 at a multiplicity of infection (MOI) of 100, and the plates were subjected to centrifugation (300 *g*, 5 min) to allow the bacterial cells to settle down. The cells were washed 2 times with warm PBS and incubated in the DMEM medium with gentamicin (50 μg/mL) for 1 h to kill the extracellular bacteria. The cells were then cultured in the DMEM medium with gentamicin (20 μg/mL). All cells were lysed by 0.25% Triton X-100 in PBS at the indicated time points, and the cell lysate was serially diluted onto the TSB plates to quantify the CFU.

**Real-time PCR analysis**

Sixteen hours prior to infection, Raw 264.7 cells were seeded into 24-well-plates at a density of 2.5 × 10^5^ cells/mL/well. The plates were incubated with Vir-2308 at a multiplicity of infection (MOI) of 100 for 4, 8, and 12 h. The cells were harvested for RNA extraction by using the RNAprep Pure cell/bacterial total RNA extraction kit (Tiangen Biotech Co., Ltd., Beijing, China), and then, cDNA was synthesized using 1 μg RNA and ReverTra Ace® qPCR RT Master Mix (Toyoba, Japan). mRNA expression levels of the housekeeping gene and target genes were quantified using SYBR Green Master mix (Vazyme Biotech Co., Ltd., Nanjing, China). The relative expression levels of the genes were determined using the 2^–∆∆Ct^ method. Table 1 presents the sequence information of primers.

**Histological analysis**

The spleens were collected from the wild-type (WT) mice or *Ifnar1*^−/−^ mice infected by Vir*-*2308 for 2 weeks. They were fixed with 4% paraformaldehyde for 24–48 h, dehydrated, and paraffin-embedded. The paraffin sections were stained with hematoxylin-eosin. The structure of the spleen and the presence of inflammation were examined.

**Flow cytometry analysis**

Tissues of spleens and mesenteric lymph nodes were collected from all mice. The single-cell suspensions of the spleens and lymph nodes were obtained by smashing all tissues and removing all cell clusters or aggregates present in the tissue solution by passing it through 40-μm cell strainers. Red blood cells were removed from splenocytes by using the 1× Red Blood Cell Lysis buffer (Tonbo Biosciences, USA). Approximately 1–2 × 10^6^ cells were used for cell surface staining and intracellular staining, and the cell surface markers were stained with specific antibodies, namely Ghost DyeTM UV450, PE-Cyanine7 anti-mouse CD45, CD4^+^ PerCP-Cyanine5.5, CD8α APC-Cyanine, F4/80^+^ APC, B220^+^Alexa Flour 700, CD3^+^ violetFlourTM 500, CD25^+^ violetFlourTM 450 CD25, CD11B^+^ BV711, Ly-6C FITC anti-mouse, Brilliant Violet 785™ anti-mouse CD11c, Brilliant Violet 650™ anti-mouse I-A/I-E, and Brilliant Violet 605™ anti-mouse NK-1.1. The transcription factor Foxp3 was intracellularly stained with the anti-mouse Foxp3 antibody (PE anti-mouse Foxp3) by using the FoxP3/Transcription Factor Staining Buffer Kit. All samples were further analyzed using a [FACSymphony A5SE](http://162.105.248.254/lims/!equipments/equipment/index.254) flow cytometer (Becton Dickinson, USA). Data were analyzed using FlowJo 10.8.0 software. The detailed information of all the antibodies is summarized in Table 2.

**Blockage of the NKG2A receptor in mice through antibody administration**

The NKG2A receptor in mice was inactivated through antibody administration. A preliminary experiment was first conducted to determine the optimal procedure of the NKG2A receptor blockage. In brief, 9 female C57BL/6 mice (6-week-old) were randomly divided into 3 groups. The 1^st^ group was administered anti-mouse NKG2A/C/E monoclonal antibody (100 µg, clone 20D5; eBioscience) through tail veil injection at 3-day intervals, the 2^nd^ group was administered the same amount of anti-NKG2A/C/E antibody at 6-day intervals, and the control group was inoculated with an equal volume of PBS by using the same strategy. The spleens and lymph nodes were collected and processed at 15 days after antibody administration, and single-cell suspensions were subjected to cell surface staining with a series of fluorochrome-conjugated antibodies, as described above, including Ghost DyeTM UV450, PE-Cyanine7 anti-mouse CD45, B220^+^Alexa Flour 700, Brilliant Ultra Violet™ 737 anti-mouse CD3, CD4^+^ PerCP-Cyanine5.5, CD8α APC-Cyanine, Brilliant Violet 605™ anti-mouse NK-1.1, and PE-anti mouse NKG2A. The blockage efficiency of NKG2A was analyzed using FlowJo 10.8.0 software. The details of all antibodies are summarized in Table 2.

For the formal blockage experiment, 24 female C57BL/6 mice (6-8 weeks old) were randomly divided into 3 groups with 8 mice in each group, control group, IgG2a isotype control group, NKG2A blockage group. Anti-mouse NKG2A/C/E monoclonal antibody was firstly administrated into each mouse using the same procedure as in the preliminary experiment followed by the *Brucella* challenge (1x10^6^/mouse) after 24 hours. The rest administration of blockage antibody was introduced into the individual mouse with 3 days intervals. The mice were euthanized 2 weeks after bacterial inoculation, and the spleens, lymph nodes, and serum were collected to determine several parameters as have described before, including CFU, histopathological changes, immune cells proportion, and inflammatory cytokines.

**Cytokine determination through ProcartaPlex multiple immunoassays**

The serum levels of 11 cytokines and chemokines, namely IFN-γ, IL-1β, IL-2, IL-4, IL-5, IL-6, IL-12 p70, IL-13, IL-18, TNF-α, and GM-CSF, were simultaneously determined using the Th1/Th2 Cytokine 11-Plex Mouse Panel (Invitrogen, EPX110-20820-901). The assay was performed and analyzed independently by Shanghai Laizee Biotech (Shanghai, China). In brief, 50 µL serum from each mouse was incubated for 2 h with a mixture of color-coded beads precoated with analyte-specific capture antibodies. After washing, biotinylated antibodies specific to the analytes were added and an antibody-antigen sandwich was formed. Then, PE-conjugated streptavidin was added to bind the biotinylated antibodies. The beads were read on a Luminex 200 analyzer. One laser was used to classify the beads and determine each analyte. The second laser was used to determine the magnitude of the PE-derived signal, which was directly proportional to the amount of analyte bound. Standard curves for these parameters were generated, and the concentration of cytokines and chemokines was determined using ProcartaPlex Analyst 1.0 software.

**Cytokine determination through ProcartaPlex multiple immunoassays**

The serum levels of 11 cytokines and chemokines, namely IFN-γ, IL-1β, IL-2, IL-4, IL-5, IL-6, IL-12 p70, IL-13, IL-18, TNF-α, and GM-CSF, were simultaneously determined using the Th1/Th2 Cytokine 11-Plex Mouse Panel (Invitrogen, EPX110-20820-901).

**RNA-seq**

WT Raw 264.7 cells or *ifnar1*^-/-^ Raw 264.7 cells were infected by Vir-2308 for 24 hours with MOI 100. RNA was extracted by Trizol followed by RNA-seq provided by Majorbio Bio-pharm Technology Co. Ltd (Shanghai, China). The RNA-seq data was analyzed on the Majorbio Cloud platform (www.majorbio.com). The software used for differential expression analysis was DESeq2. A gene was considered a differentially expressed gene (DEG) if it met the criteria of |log2FC|≥1 and FDR < 0.05. For the subsequent analysis, GO enrichment analysis was performed using the software Goatools, employing Fisher's exact test. A GO term was considered significantly enriched if its adjusted p-value (p_fdr) was < 0.05.

**Results**

**Vir-2308 infection elicits strong immune responses in mice compared with Vac-A19 infection**

In DCs, the GO analysis demonstrated upregulated signaling pathways, such as autophagy, mitophagy, extrinsic apoptotic signaling pathway, I-kappaB kinase/NF-kappaB signaling, antigen processing and presentation, phagocytosis, T cell differentiation, type II IFN production, leukocyte chemotaxis, regulation of interleukin-6 production, and endosome to lysosome transport in Vir-2308 infected and Vac-A19 infected groups (Fig. 3A, Supplementary File 7). Vir-2308 induced upregulation of signaling pathways in Mϕ (e.g., autophagy, response to type II IFN, response to oxidative stress, oxidative phosphorylation, ncRNA processing, phagocytosis, I-kappaB kinase/NF-kappaB signaling, neutrophil migration, TNF production, interleukin-1 beta production, necrotic cell death, regulation of the NLRP3 inflammasome complex assembly, ERAD pathway, type I IFN production, lipopolysaccharide-mediated signaling pathway, and NK T cell differentiation) (Fig. 3B, Supplementary File 7). The GO analysis revealed that Vir-2308 induced the upregulation of several immune-relevant signaling pathways, such as autophagy, response to oxidative stress, response to endoplasmic reticulum stress, Wnt signaling pathway, regulation of stress-activated MAPK cascade, and cellular response to type II interferon compared that in Vac-A19 group (Fig. 3C, Supplementary File 7).

**Immune cell landscapes in murine lymph nodes**

Compared with the Ctrl group (0.23%), relative ratios of DCs increased at 14 dpi in the Vir-2308 group (1.07%). By contrast, a relatively higher ratio of DCs was observed in the Vir-2308 group (0.37%). Nφ ratios were elevated in the Vir-2308 group (0.18%) compared with the Ctrl group (0.06%) at 14 dpi (Fig. 2A). Moreover, compared with the percentage of total T cells in the Ctrl group (77.51%), the percentage of total T cells reduced in the Vir-2308 group (53.09%) at 14 dpi. (Fig. 2A). A further analysis with T cell subtypes revealed that the ratio of CD4^+^ T cells (38.68%) and CD8^+^ T cells (14.41%) decreased in the Vir-2308 group at 14 dpi compared with the Ctrl group, respectively (53.00%, 24.51%).

Consistent with majority of the scRNA-seq data, the flow cytometry analysis obtained from 14 dpi revealed that Vir-2308 infection drastically increased the relative ratio of B cells by almost 2-fold compared with that in the Ctrl group. Inflammatory monocytes and Nφ also significantly increased in the Vir-2308 group. The proportions of T cells and MHC II^+^ DCs reduced in the Vir-2308 group compared with the Ctrl group. Vir-2308 infection caused a distinct NK cell loss at the early stage of *Brucella* infection (Fig. 2B).

**Immune responses and inflammatory responses are activated in certain innate immune cells in response to *Brucella* infection**

GO and KEGG analyses unveiled that significantly enriched pathways primarily included oxidative phosphorylation, T cell receptor, endocytosis, ubiquitin-mediated proteolysis, and proteasome signaling pathways in the Mϕ, Nφ, DCs, and NK cells in the lymph nodes of the Vir-2308 group at 14 dpi compared with the Ctrl group (Supplementary Files 2-5). The analysis unveiled that type I and type II interferon (IFN) production, tumor necrosis factor (TNF) production, Toll-like receptor (TLR) signaling pathway, phagocytosis, necrotic cell death, mitochondria respiratory chain complex assembly, JNK cascade, cell apoptosis, endosome to lysosome transport, and autophagy were activated in the DCs in the Vir-2308 groups at 14 dpi and 90 dpi (Fig. 3A). According to further analysis of the enriched signaling pathways as per GO terms, Mϕ in the Vir-2308 groups exhibited a relatively higher number of induced signaling pathways than those in the Vac-A19 group at 14 dpi (e.g., autophagy, phagocytosis, type I IFN, and TLR signaling pathways; Supplementary File 6).

**IFN signaling pathways, cell death pathways, and certain immune signaling pathways were activated in mice in response to *Brucella* infection**

Regarding cell death signaling pathways, typical genes (*Atg3*, *Atg5*, *Atg12*, *Atg16*, *p62*, *LC3*, *Ulk1*, *Casp3*, *Casp8*, *Casp9*, *Fadd*, *Ripk3*, *Mlkl*, *Casp1*, *Casp11*, *GSDMD*, *GPX4*, *Nos*, and *Alox*) associated with several cell death types (e.g., apoptosis, autophagy, necroptosis, pyroptosis, and ferroptosis) were selected. Our scRNA-seq data unveiled that *Atg12*, *Ulk1*, and *Casp3* were upregulated in the Vir-2308 group Moreover, cell death-associated genes were induced at relative higher levels in the lymph nodes at the early stage (14 dpi) of infection compared with the later stage (90 dpi) of infection (Supplementary File 8C).

The CellPhone DB analysis revealed strong interactions between DCs vs Mϕ, NK cells vs CD4^+^ T cells, NK cells vs Mϕ, Nϕ vs monocytes, and NK cells vs CD8^+^ T cells (Fig. 3D). In brief, CD74_APP, CD74_COPA, and CD74_MIF are active during the interaction between DCs, Mϕ, B cells, and other cell types (Fig. 3D). Further analysis with DEG in CD4^+^ T cell and CD8^+^ T cells in the lymph nodes from Vir-2308 group at 14 dpi did not observe the up-regulation of NKG2A (Date not shown).

**Type I IFN signaling contributes to *Brucella* infection pathogenesis**

Fig 3C presents the workflow experimental setup. Two WT mice infected with Vir-2308 were unexpectedly dead after the bacterial challenge most likely due to the issue of individual difference in the mice (Fig. 4D). Significant splenomegaly was present in the *Brucella*-infected WT mice and *Brucella*-infected *Ifnar1*^–/–^ mice (Fig. 4E).A clear boundary was observed between the red pulp and white pulp in the control group. Similar levels of histopathological changes, for example, disrupted spleen structure, no clear boundary between the red pulp and white pulp, and infiltration with inflammatory cells (mainly lymphocytes and monocytes) in the red pulp, were observed in the spleens of the Vir-2308-infected WT mice and Vir-2308-infected *Ifnar1*^−/−^ mice (Fig. 4F).

Compared with the Vir-2308-infected WT mice, the Vir-2308-infected *Ifnar1*^−/−^ mice exhibited significantly elevated ratios of Mϕ (*p* < 0.05) and NK cells (*p* < 0.01). An increased trend of MHC II^+^ DCs (*p* = 0.072) and Nϕ (*p* = 0.057) was also observed in the lymph nodes (Fig. 4G, 4I). A similar relative proportion of the examined 11 immune cell types was observed in the spleens from the *Brucella* infected-WT mice and *Brucella* infected-*Ifnar1*^−/−^ mice (Fig. 4H).

**Legend of Supplementary Figures**

**Supplementary File 7. Characterization of host signaling pathways in response to *Brucella* infection.** L14_2308 stands for the lymph node samples from mice infected by *Brucella* 2308 for 14 days, S14_A19 strands for spleen samples from mice infected by Brucella A19 for 14 days, and the names for the other groups follows the same rule. A. Shared hallmark signaling pathways in DCs in the lymph nodes from mice infected by Vir-2308 or Vac-A19 at 14 dpi and 90 dpi (termed as L14_2308, L90_2308, L14_A19, L90_A19, respectively) were exhibited with bubble diagrams. B. Common hallmark signaling pathways in Mϕ in the lymph nodes from infected by Vir-2308 or Vac-A19 at 14 dpi and 90 dpi (termed as L14_2308, L90_2308, L14_A19, L90_A19, respectively). C. Common hallmark signaling pathways in NKs in the lymph nodes from mice infected by Vir-2308 or Vac-A19 at 14 dpi and 90 dpi (termed as L14_2308, L90_2308, L14_A19, L90_A19, respectively).

**Supplementary File 8. Characterization of host genetic responses to *Brucella* infection.** There are 12 groups in the scRNA-seq experimental setup. L14_2308 stands for the lymph node samples from mice infected by *Brucella* 2308 for 14 days, S14_A19 strands for spleen samples from mice infected by Brucella A19 for 14 days, and the names for the other groups follows the same rule. **A.** The dot plot showed the relative expression profile of type I IFN (IFN-α, IFN-β), type II IFN (IFN-γ), and IFN receptors (*ifnar1*, *ifngr1*, *ifnar2*, *ifngr2*) in the individual cell type (B cells, T cells, CD4^+^ T cells, CD8^+^ T cells, Cycling cells, Mϕ, IM, Nϕ, DCs, NKs). **B.** The dot plot showed the relative expression profile of IFN receptors (*ifnar1*, *ifngr1*, *ifnar2*, *ifngr2*) and type I IFN-related genes (*irf3*, *irf7*, *irf9*) in all experimental groups. **C.** Expression levels of typical cell death-related host genes (*atg3*, *atg5*, *atg12*, *Casp3*, *Casp8*, *Casp9*, *Ulk1*, *Tfeb*, *Fadd*, *Ripk3*, *Mlkl*, *Casp1*, *Gsdmd*) in all experimental groups. **D.** IFN-β expression in 9 types of cells (B cells, CD4^+^ T cells, CD8^+^ T cells, Mϕ, Cycling cells, Nϕ, DCs, NKs, NKT) examined. **E.** Expression of type I IFN (IFN-α, IFN-β) and **F-K.** type II IFN (IFN-γ) of all experimental groups in all cell types (F), in Mϕ (G), in NK cells (H), in NKT cells (I), in CD4^+^ T cells (J), and in CD8^+^ T cells (K). Mϕ: macrophages; DC: dendritic cells; NKs: natural killer cells; NKT: natural killer T cells;

**Supplementary File 11. RNA-seq of wild-type Raw 264.7 cells or *ifnar1*^-/-^ Raw 264.7 cells infected by Vir-2308.** The volcano histogram (Left) presented the up-regulated, down-regulated and unchanged genes in wild-type Raw 264.7 cells or *ifnar1*^-/-^ Raw 264.7 cells infected by Vir-2308 at 24 hours post infection. Compared to the wild-type Raw 264.7 cells infected by *Brucella,* some enriched signaling pathways induced in *ifnar1*^-/-^ Raw 264.7 cell as per GO terms were shown. (Fight). KOS24: *ifnar1*^-/-^ Raw 264.7 cells infected by Vir-2308 at 24 hours post infection; WTS24: wild-type Raw 264.7 cells infected by Vir-2308 at 24 hours post infection.
