# Supplementary figures and images for: Single-Cell Transcriptome Profiling Reveals the Immune Dysregulation Characteristics of Mice Infected With Brucella abortus
Source: J Infect Dis. 2025 Oct 11;233(1):e55–66. doi: 10.1093/infdis/jiaf522 (PMC12811882; doi:10.1093/infdis/jiaf522)

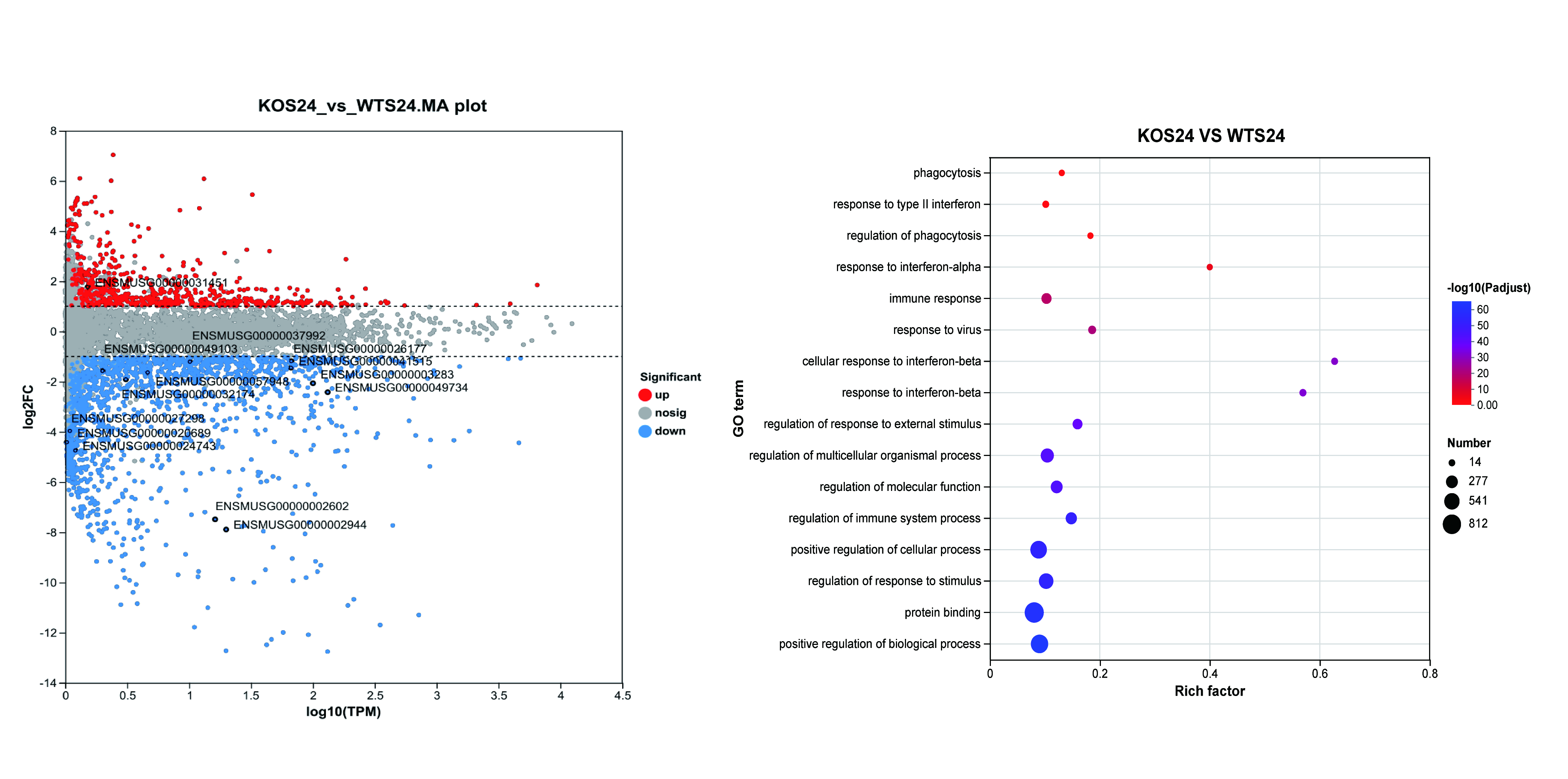

Supplement: jiaf522_Supplementary_Data [file jiaf522_supplementary_data.zip › Supplementary File 11_IFNAR KO Marophage RNA-seq-07.tif]

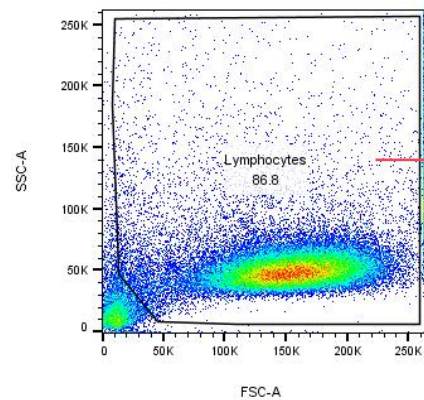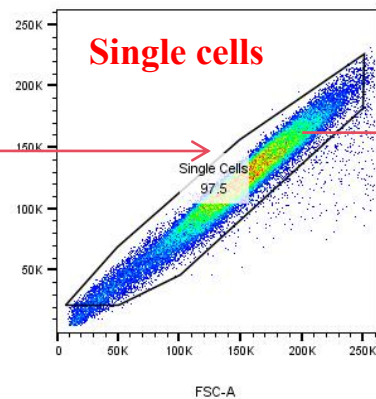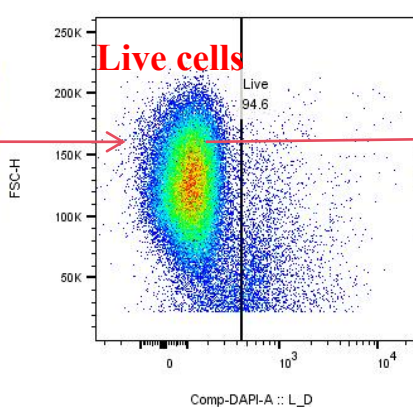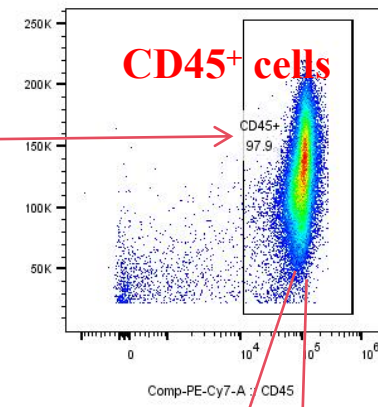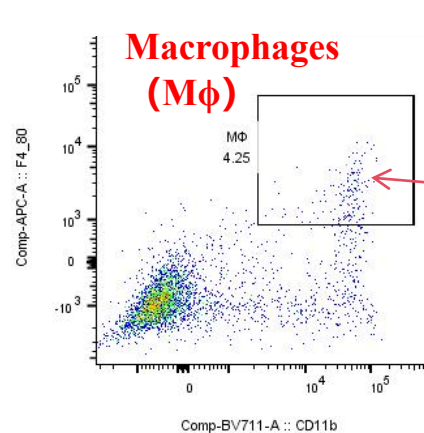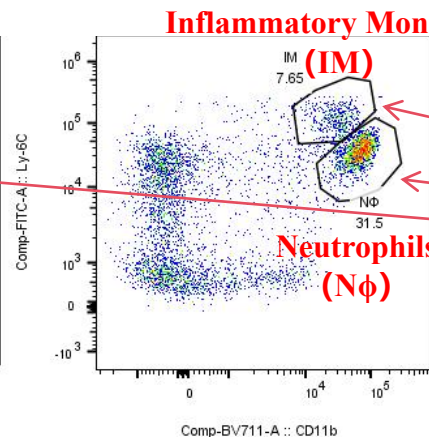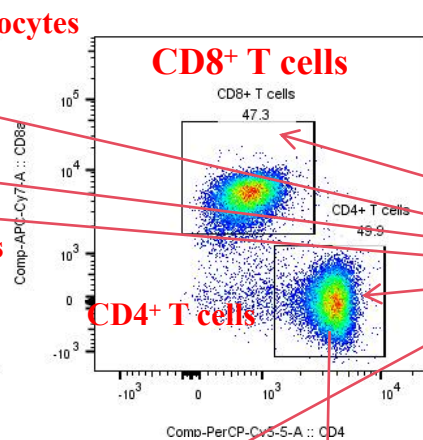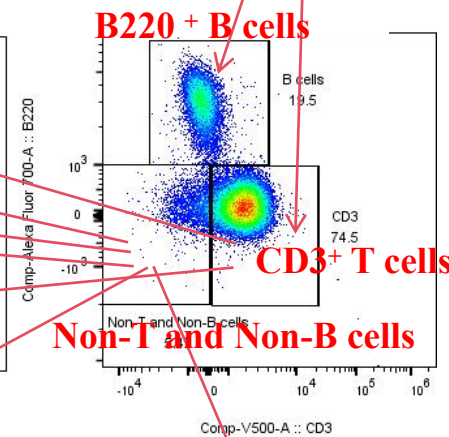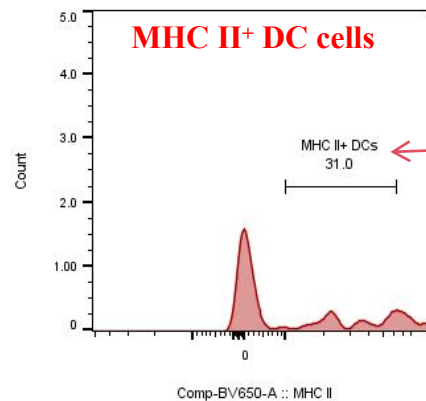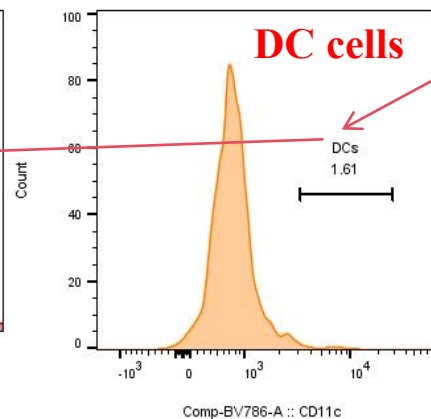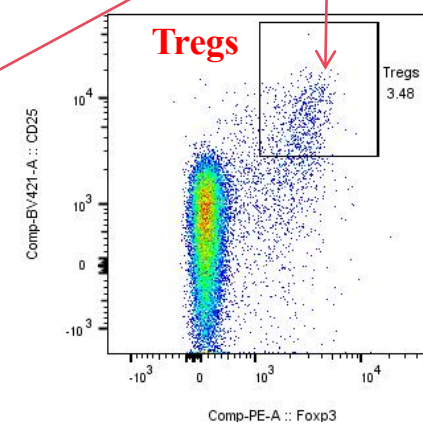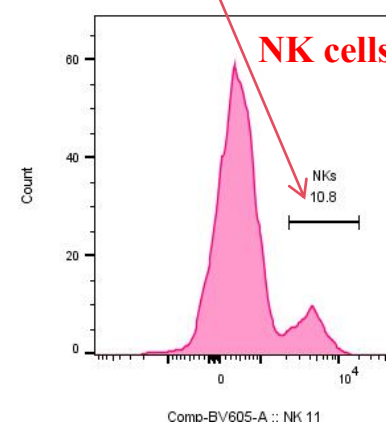

Supplement: jiaf522_Supplementary_Data [file jiaf522_supplementary_data.zip › Supplementary File 1_Flow Cytometry Gating Strategy.pdf]

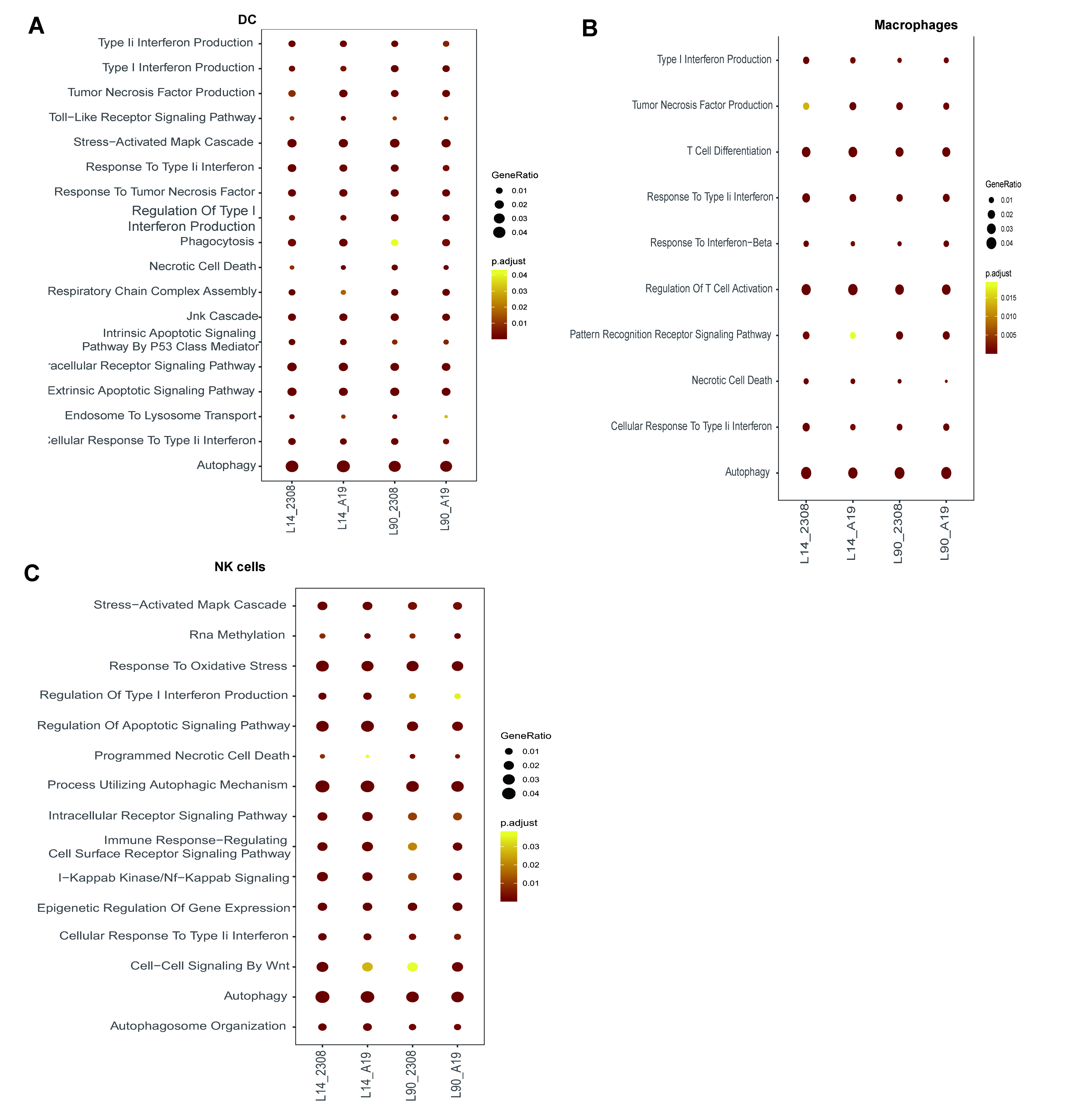

Supplement: jiaf522_Supplementary_Data [file jiaf522_supplementary_data.zip › Supplementary File 7_DC, Macrophages, NKs GO up 4 groups.tif]

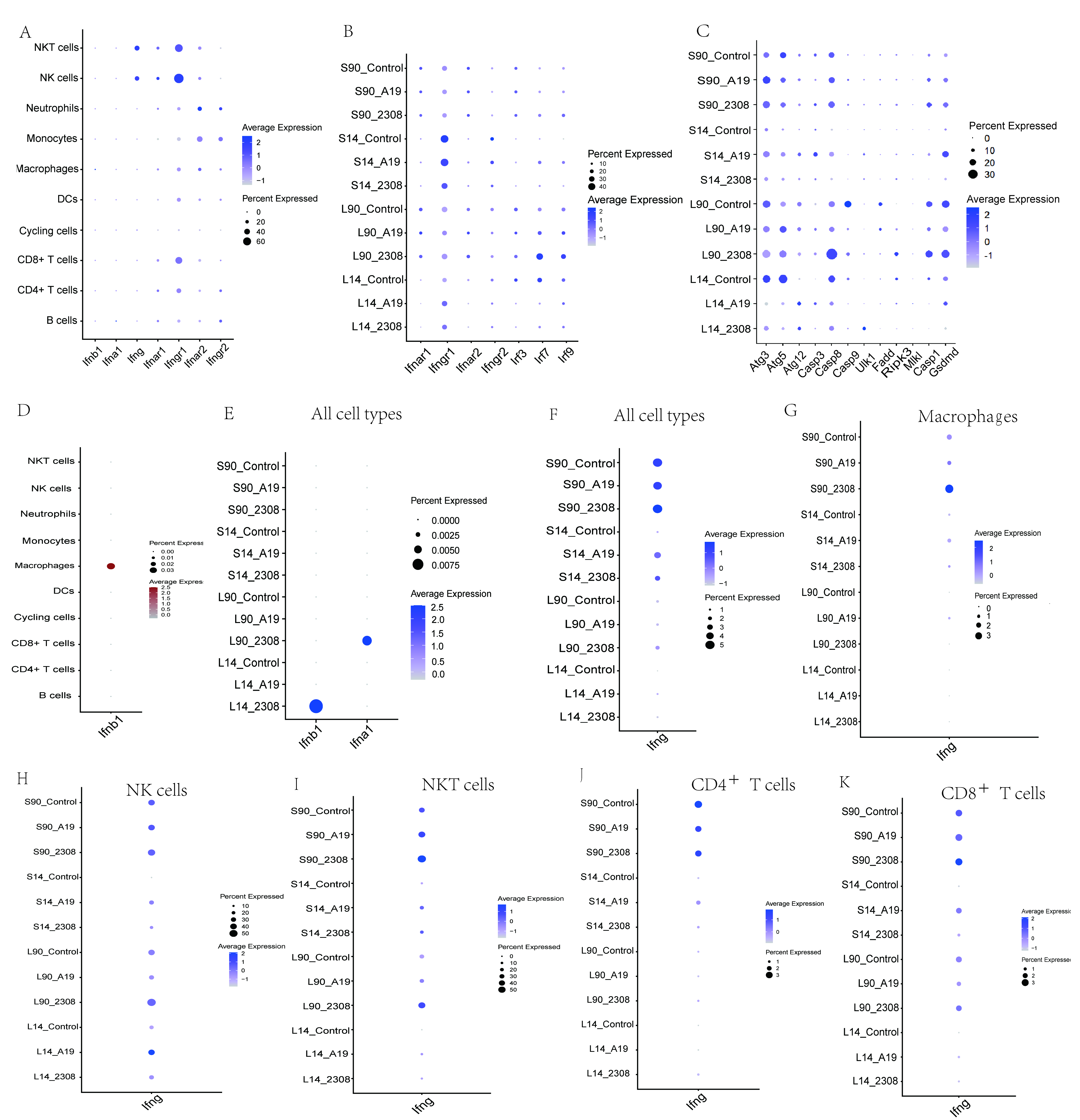

Supplement: jiaf522_Supplementary_Data [file jiaf522_supplementary_data.zip › Supplementary File 8 (Revised)_IFNs and Cell death genes.tif]

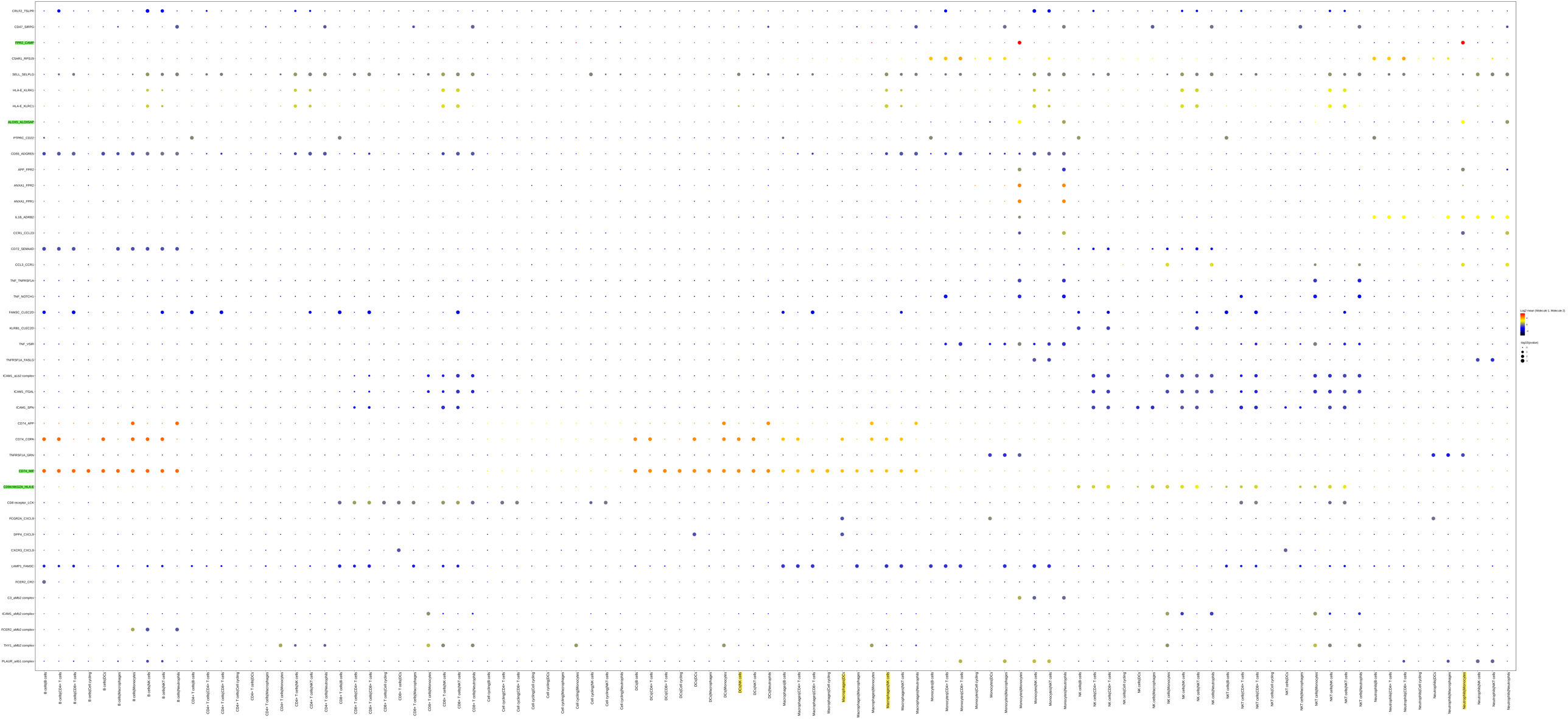

Supplement: jiaf522_Supplementary_Data [file jiaf522_supplementary_data.zip › Supplementary File 9_Cell communciation.pdf]
